# Transdermal administration of farnesol-ethosomes enhances the treatment of cutaneous candidiasis induced by *Candida albicans* in mice

**DOI:** 10.1128/spectrum.04247-23

**Published:** 2024-02-28

**Authors:** Ting Shen, Baocheng Tian, Wei Liu, Xuesong Yang, Qi Sheng, Mengxin Li, Haiyan Wang, Xiuwen Wang, Huihui Zhou, Yanchun Han, Chen Ding, Sixiang Sai

**Affiliations:** 1 School of Pharmacy, Binzhou Medical University, Yantai, Shandong, China; 2 College of Life and Health Science, Northeastern University, Shenyang, China; 3 Department of pathology, Affiliated Yuhuangding Hospital of Qingdao University, Yantai, Shandong, China; 4 Department of Pathology, Binzhou Medical University, Yantai, Shandong, China; University of Debrecen, Debrecen, Hungary

**Keywords:** farnesol, ethosomes, cutaneous candidiasis, morphogenesis, antifungal activity, drug delivery efficacy

## Abstract

Cutaneous candidiasis, caused by *Candida albicans*, is a severe and frustrating condition, and finding effective treatments can be challenging. Therefore, the development of farnesol-loaded nanoparticles is an exciting breakthrough. Ethosomes are a novel transdermal drug delivery carrier that incorporates a certain concentration (10–45%) of alcohols into lipid vesicles, resulting in improved permeability and encapsulation rates compared to conventional liposomes. Farnesol is a quorum-sensing molecule involved in morphogenesis regulation in *C. albicans*, and these ethosomes offer a promising new approach to treating this common fungal infection. This study develops the formulation of farnesol-loaded ethosomes (farnesol-ethosomes) and assesses applications in treating cutaneous candidiasis induced by *C. albicans in vitro* and *in vivo*. Farnesol-ethosomes were successfully developed by ethanol injection method. Therapeutic properties of farnesol-ethosomes, such as particle size, zeta potential, and morphology, were well characterized. According to the results, farnesol-ethosomes demonstrated an increased inhibition effect on cells’ growth and biofilm formation in *C. albicans*. In Animal infection models, treating farnesol-ethosomes by transdermal administration effectively relieved symptoms caused by cutaneous candidiasis and reduced fungal burdens in quantity. We also observed that ethosomes significantly enhanced drug delivery efficacy *in vitro* and *in vivo*. These results indicate that farnesol-ethosomes can provide future promising roles in curing cutaneous candidiasis.

**IMPORTANCE:**

Cutaneous candidiasis attributed to *Candida* infection is a prevalent condition that impacts individuals of all age groups. As a type of microbial community, biofilms confer benefits to host infections and mitigate the clinical effects of antifungal treatments. In *C. albicans*, the yeast-to-hypha transition and biofilm formation are effectively suppressed by farnesol through its modulation of multiple signaling pathway. However, the characteristics of farnesol such as hydrophobicity, volatility, degradability, and instability in various conditions can impose limitations on its effectiveness. Nanotechnology holds the potential to enhance the efficiency and utilization of this molecule. Treatment of farnesol-ethosomes by transdermal administration demonstrated a very remarkable therapeutic effect against *C. albicans* in infection model of cutaneous candidiasis in mice. Many patients suffering fungal skin infection will benefit from this study.

## INTRODUCTION

The prevalence of clinical *Candida* infection remains high due to the increasing use of immunosuppressants, organ transplantation, and antibiotics in cancer chemotherapy. Cutaneous candidiasis is a common skin disease caused by *Candida* species that can affect patients of all ages (1, 2). This disease accounts for approximately 1% of outpatients and 7% of dermatological clinic patients (3). Patients may experience candidiasis in all body regions, including intertrigo, cheilitis, diaper dermatitis, and interdigital candidiasis. *Candida albicans* is the most prevalent among all candidiasis-causing fungi (4, 5).

*C. albicans* is known for its highly versatile adaptability to the host environment, achieved by switching between budding yeast cells and filamentous pseudohyphal and hyphal cells (6). Its morphogenesis regulation in response to the host environment is essential to its virulence. Once it adheres to host surfaces like mucosal surfaces, epithelial cell linings, and parenchymal organs, yeast cells transit to hyphae cells, and biofilms initiate formation (7, 8). Biofilms help increase adherence to host surfaces, enhance the establishment of infections in the hosts, and protect pathogens from host defenses and drugs (9). As a form of microbial communities, biofilms benefit pathogens with spatial stability and independence by governing their microenvironment (10). Biofilms in *C. albicans* are arranged in a bilayer structure containing large amount of yeast and hyphal cells (11). *C. albicans* biofilms also employ a range of mechanisms to tolerate antifungals available for clinical application, and these mechanisms differ depending on the stages of biofilm formation (12, 13). Therefore, developing novel antifungals with more activities in this microenvironment is crucial. As a result, the search for potential antifungals that inhibit the formation of biofilms in *C. albicans*, such as farnesol, has attracted considerable attention (14).

Farnesol is a byproduct of sterol biosynthesis and acts as a quorum-sensing molecule (QSM) in *C. albicans* (15, 16). Quorum sensing is a unique mechanism by which microorganisms sense their population density and regulate gene expression in response to accumulated QSM in the extracellular environment. This phenomenon promotes cell-to-cell communication to synchronize single cells as multicellular organisms (17, 18). In *C. albicans*, farnesol significantly inhibits the yeast-to-hypha transition and biofilm formation by targeting several signaling pathways, such as hyphal initiation and maintenance (18, 19). Virulence-associated characteristics of *C. albicans* influenced by farnesol make it a potential novel antifungal drug (20). Farnesol is also known to have inhibitory effects on other fungi and bacteria, such as *Aspergillus niger*, *Aspergillus fumigatus*, *Candida parapsilosis*, *Candida dubliniensis*, and *Staphylococcus epidermidis* (21–25). The trait of farnesol is highly hydrophobic and volatile, and many factors, such as high temperatures, humidity, light, and oxygen, cause farnesol degradation (26). As a result, the hydrophobicity and instability of farnesol limits its bioavailability as a potential antifungal drug against *C. albicans*. Although topical use of farnesol is generally safe, it can cause skin irritation due to allergic reactions (26). Nanoparticles might be an effective tool to improve farnesol bioavailability with the development of nanotechnology.

Cutaneous candidiasis is currently treated with topical and oral therapies with anti-inflammatory, antibacterial, and antifungal effects (27). Transdermal drug delivery is more moderate for therapeutic agent administration than other routes. This method can improve medication bioavailability by designating medicines in a specific skin region and decreasing the possibility of adverse effects (28). Additionally, functional nanoparticles offer an attractive solution for transdermal drug delivery. These nanoparticles have unique physicochemical features, such as nanoscale effect, form, stiffness, thermo-responsiveness, surface charge, and pH responsiveness (29–32). These intrinsic features affect skin penetration and improve the transdermal capacity by interacting with biological molecules, making nanoparticles enhance skin penetration efficacy better than a skin surface reservoir (33). Among these nanoparticles, ethosomes containing higher concentration alcohols such as ethanol and isopropyl alcohol form lipid vesicles with enhanced permeability and encapsulation rate, and show excellent stability and skin tolerance (34). Therefore, whether farnesol-loaded nanoparticles can treat cutaneous candidiasis caused by *C. albicans* is worth considering.

This study aims to investigate using ethosomes as a nanocarrier for farnesol entrapment. The study evaluated therapeutic properties such as particle size, zeta potential, and farnesol-ethosome entrapment efficiency. We assessed the antifungal activity of farnesol-ethosomes against yeast cells in *C. albicans in vitro* using the minimum inhibitory concentration. The matured biofilm in response to farnesol-ethosomes was then determined in *C. albicans* cells. The study also determined the expression pattern of genes involved in dimorphic switching control in response to farnesol-ethosomes. Moreover, we constructed an animal model of cutaneous candidiasis to determine the effect of farnesol-ethosomes against *C. albicans* cells in mice. Then, coumarin 6-ethosomes were developed to evaluate the effect of ethosomes on drug delivery efficacy in *C. albicans* cells and animal models. We also assessed farnesol-ethosome cytotoxicity in mammalian cells and animal models. Our data demonstrate *in vitro* that these novelty farnesol-ethosomes are active against *C. albicans* cells. The ethosome delivery systems demonstrated higher drug delivery efficacy compared to traditional therapy *in vitro* and *vivo*. Farnesol-ethosomes also showed optimistic results in the treatment of cutaneous candidiasis induced by *C. albicans*, suggesting excellent prospects in treating cutaneous candidiasis.

## MATERIALS AND METHODS

### Preparation of farnesol-ethosomes

The farnesol-loaded ethosomes were developed using the ethanol injection method as described in reference (35). Specifically, 8 mg soy phospholipids and 2 mg cholesterol were weighed and dissolved in 0.2 mL anhydrous ethanol. The ethanol solution was injected slowly into 0.2 mL phosphate-buffered solution (PBS, pH 6.8) at the rate of 200 µL/min. About 10 µL farnesol solution (0.4 M) was mixed thoroughly into the ethanol solution under ultrasonic conditions by a Scientz-II D probe sonicator (Ningbo Scientz Biotechnology Co., LTD, Zhejiang, China) at 100 W for 3 min in an ice bath. The prepared farnesol-ethosomes solution was injected into 0.6 mL of deionized water under ultrasonic conditions. The obtained farnesol-ethosomes (4 mM) were stored at 4°C for further research. The preparation of C6 loaded ethosomes was identical to the above procedure, except that C6 solution was added into ethanol solution instead of farnesol solution.

### Physicochemical characterization of farnesol-ethosomes properties

The nanoparticle size and zeta potential were detected by the dynamic light scattering using a Malvern ZEN3600 Zetasizer (Malvern Instruments, UK) after appropriate dilution. Each measurement was carried out in triplicate at room temperature. The entrapment efficiency of farnesol in the ethosomes was determined indirectly by the ultrafiltration method as reported previously. First, the ethosome sample was added to the upper chamber of an Ultra centrifugal filter device (Millipore Corporation, USA), and centrifuged for 25 min at 5000 rpm (Shanghai Anting TDL-60B, China) to separate nanoparticles from the aqueous suspension medium. The unentrapped farnesol in the filtrate was quantified by high-performance liquid chromatography method. The entrapment efficiency (EE%) and drug loading (DL%) could be calculated from the following equations:


$$EE\%=\frac{W_{total}-W_{free}}{W_{total}}\times 100$$

$$DL\%=\frac{W_{total}-W_{free}}{W_{total}-W_{free}+W_{lip}}\times 100$$

where *W*_total_ is the total drug amount added for ethosome preparation, *W*_lip_ is the total lipid materials amount used for ethosome preparation, and *W*_free_ is the amount of free drug determined in the filtrate. The morphology of the nanoethosomes was observed using transmission electron microscopy (TEM), and the TEM photographs were obtained by JEM 1400 (JEOL, Japan). Briefly, one drop of ethosome sample was dropped onto a 200-mesh copper grid covered with carbon film and drained with filter paper. As a negative stain, 1.5% phosphotungstic acid was administered for 20 min and dried naturally for 24 h for observation. The stability against storage of farnesol-loaded ethosomes was evaluated by measuring changes in particle size and zeta potential during storage at 25°C.

### Antifungal activity of farnesol-ethosomes *in vitro*

The antifungal activity of farnesol-ethosomes and farnesol *in vitro* was directed by the guidelines of the National Committee for Clinical Laboratory Standards. *C. albicans SC5314* was grown overnight in yeast extract peptone dextrose (YPD) medium at 30°C. Serial dilutions of tested compound were 800–100 µM in YPD medium. Compounds were dispensed, respectively, into 96-well plate by pipette. An additional 100 µL YPD medium containing 1 × 10^4^ CFU *C*. *albicans* cells was added to each well. The identical quantity of *C. albicans* cells was added to 200 µL YPD medium as the control group. The 96-well plates were then incubated at 30°C for 12 h at 200 rpm. Finally, the optical density of *C. albicans* cells was determined by microplate spectrophotometry at 600 nm (Thermo-Fisher Scientific, Waltham, MA, USA). The cell viability rate was calculated as a percentage of *C. albicans* cell growth with tested compounds compared to the control (no drug).

### Biofilm growth of *C. albicans* in response to farnesol-ethosomes

*C. albicans SC5314* was grown overnight in YPD medium at 30°C and then diluted in Spider medium to 5 × 10^7^ CFU/mL. Squares of silicone (1.5 × 1.5 cm^2^) were prepared and incubated with bovine serum (Sigma-Aldrich) overnight in advance. Squares washed with 2 mL PBS buffer were transferred to a 12-well plate. About 2 mL of *C. albicans* cells in Spider medium was added to each well containing silicon squares. In order to initiate the adhesion of biofilm growth, the plate was incubated at 37°C for 1.5 h. Squares were carefully collected and gently washed with PBS. After this step, squares were relocated into new wells containing 3 mL of fresh Spider medium. The biofilm growth model of *C. albicans* was matured after incubation at 37°C for 48 h. Spider medium containing tested compound was prepared and added into each well to reach final concentrations 100, 200, and 800 µM individually. All plates were incubated at 37°C for an additional 24 h. The results were photographed by confocal microscopy (Leica TCS SPE). Concanavalin A–Alexa Fluor 594 conjugate excitation was generated through UV light at less than 590 nm. Emission profiles were then collected at 618 nm.

### Quantitative real-time PCR

The gene expression level in response to farnesol-ethosomes and farnesol treatment in *C. albicans* was determined by real-time PCR. *C. albicans SC5314* was grown overnight in YPD medium at 30°C and then diluted in Spider medium to 2 × 10^6^ CFU/ml. *C. albicans* cells were treated with 400 µM farnesol-ethosomes and farnesol individually and incubated at 37°C for 3 h. *C. albicans* cells were harvested and washed two times with PBS buffer and then total RNA in each group was extracted by RiboPure kit (Thermo-Fisher). Then, cDNA was synthesized using SuperScript VILO kit (Thermo-Fisher Scientific, Waltham, MA, USA) as performed by the manufacturer’s protocol. Primers were designed using Primer 3.0plus (http://www.bioinformatics.nl/cgi-bin/primer3plus/primer3plus.cgi), and *ACT1* was used as an endogenous control. The primers used are shown in Table 1. For real-time PCR, 6.25 µL of 2× SYBR green master mix (Agilent), 4.75 µL of water, and 1 µL of each oligonucleotide (5 µM) were mixed with 0.5 µL of cDNA. Samples were run in duplicate. The PCR program was carried out on a Stratagene Mx3005P (Thermo-Fisher Scientific, Waltham, MA, USA) and consisted of denaturing and activation steps at 95°C for 10 min, followed by 30 cycles of 95°C for 15 s, 55°C for 30 s, and 72°C for 30 s. ΔCt values were calculated by subtracting Ct for *ACT1* from the C*t* of the gene of interest. Relative expression was calculated from the ratio of 2^−Δ C*t*^ for the test condition relative to 2^−Δ C*t*^ for the control condition. Standard deviations were calculated from the replicates and divided by the value for 2^Δ C*t*^ for the control group.

**TABLE 1:** List of primers used in quantitative real-time PCR

Primer name	Sequence e (5′ to 3′)
ACT1-F	GCGGTAGAGAGACTTGACCAACC
ACT1-R	GACAATTTCTCTTTCAGCACTAGTAGTG
HWP1-F	ACTGCTCAACTTATTGCTATCGC
HWP1-R	ACCGTCTACCTGTGGGACAG
ALS3-F	TGCCGGTTATCGTCCATTTG
ALS3-R	AGGTGCACGTTGCCAATAAC
RAS1-F	ACTAGTGCTGTTAATGGTGGTG
RAS1-R	TGCTTGACCAGAAGAAACACC
EED1-F	AACGTCATCAACCGTTGCAG
EED1-R	TGTTTGTGGTTGCAGCAGTG
UME6-F	TCTGGAGTTGGGACTAGGATTG
UME6-R	TGGTGTTGGTTGGGATTGTG

### Antifungal activity of farnesol-ethosomes *in vivo*

Instructions (NIH Publications No. 8023, revised 1978) were carefully followed in animal experiments involving in this study. Research Ethics Committees of Binzhou Medical University (No. 2021-301) reviewed and approved all animal experiments. Male Kunming mice (4–5 weeks, 13–16 g) were acquired from Jinan Pengyue Experimental Animal Breeding Co., LTD (JPEXABC) (Jinan, Shandong, China). The protocol of subcutaneous infection was performed as described elsewhere (36). Twenty-four mice were randomly chosen and divided (*n* = 6) into four groups. Four groups of mice were anesthetized and infected subcutaneously with 200 µL sterile PBS containing a certain amount of *C. albicans* cells. At 3 days post-infection, mice in each group received respectively PBS, 400 µM farnesol, 400 µM farnesol-ethosomes, and 4 mM farnesol-ethosomes treatment with a volume of 10 µL by transdermal administration daily. Day 1 was considered as the treatment initiation. Treatments in four groups lasted until Day 10. All mice were photographed daily by a camera. All mice were sacrificed at Day 10. Partial skin in lesion sites was then isolated, weighed, and placed in 10 mL PBS. Next, tissues were homogenized, and the suspension was plated on YPD agar at 30°C for 2 days. The number of visible colony-forming units (CFUs) was carefully counted by clicker.

### C6-ethosomes uptake efficacy *in vitro* and *in vivo*

The assay of ethosomes uptake efficacy was determined using coumarin fluorescent ethosomes. Coumarin 6 (C6) was encapsulated into ethosomes. The characteristics of C6-ethosomes were examined in order to correspond with farnesol-ethosomes. The protocol of C6-ethosomes uptake efficacy in yeast cells in *C. albicans* was performed as described elsewhere (37). Yeast cells were treated with C6 -ethosomes and C6 at 30°C. At four post-treatment time points (1, 3, 6, and 9 h), 400 µL *C*. *albicans* cells in suspension was collected and then photographed by Leica TCS SPE. C6 excitation was generated through UV light at less than 470 nm. Emission profiles of C6 excitation were then collected at 490 ± 10 nm. For assay of C6-ethosomes uptake efficacy *in vivo*, 30 male Kunming mice (4–5 weeks, 13–16 g) were acquired from JPEXABC and randomly divided (*n* = 10) into three groups. Three groups were subjected to treatment using C6-ethosomes (20 µg/mL), C6 (20 µg/mL), and PBS buffer with a volume of 10 µL. Treatment with C6-ethosomes, C6 and PBS buffer by transdermal administration were performed in three groups. At two post-treatment time points (6 and 24 h), five mice in each group were sacrificed and partial skin with test compound treatment were then isolated. Skin tissues in all groups were examined by Leica TCS SPE. Five fluorescent images in each group were analyzed by ImageJ (National Institutes of Health). A mean gray value of C6 fluorescence intensity was achieved through total intensity among cells divided by cell area in each image.

### Cytotoxicity assay of farnesol-ethosomes *in vitro* and *in vivo*

WST assay was used for evaluating the cytotoxicity of the farnesol-ethosomes *in vitro*. The human embryonic kidneys cell line 293T was cultured in 500 mL of complete cell culture medium (DMEM) supplemented with 10% fetal bovine serum at 37°C with 5% CO_2_ for 24 h. 293T cells were harvested at logarithmic growth stage and then digested by trypsin treatment. The cell density was adjusted to 5 × 10^4^ cells/mL in DMEM medium. 293T cells were treated with DMEM medium containing different farnesol-ethosomes concentrations (10–400 µM) individually in each well. The 96-well plate was cultured at 37°C with 5% CO_2_ for 24 h. The quantity of viable cell number was determined by Cell Counting Kit-8 (Sigma, Cleveland, OH, USA) as performed by the manufacturer’s protocol. Cell viability rate was calculated as a percentage of vehicle control using GraphPad Prism v.9.0 (GraphPad, San Diego, CA, USA). Pathological section assay was used for evaluating the cytotoxicity of the farnesol-ethosomes *in vivo*. Fifteen male Kunming mice (4–5 weeks, 13–16 g) were acquired from JPEXABC and randomly divided (*n* = 5) into three groups. Treatment with 10 µL PBS, 400 µM farnesol, and 400 µM farnesol-ethosomes by transdermal administration was performed in three groups daily. At 10 post-treatment day, all mice were sacrificed and skin tissues treated with test compounds were dissected and stained with hematoxylin and eosin (H&E) for pathological section.

### Statistical analysis

Experiments were independently repeated at least three times for reproducibility. Statistical analyses were performed using the GraphPad Prism v.9.0. Statistically significant differences were set at *P* ≤ 0.05.

## RESULTS

### Characterization of farnesol-ethosomes

**Fig 1:**
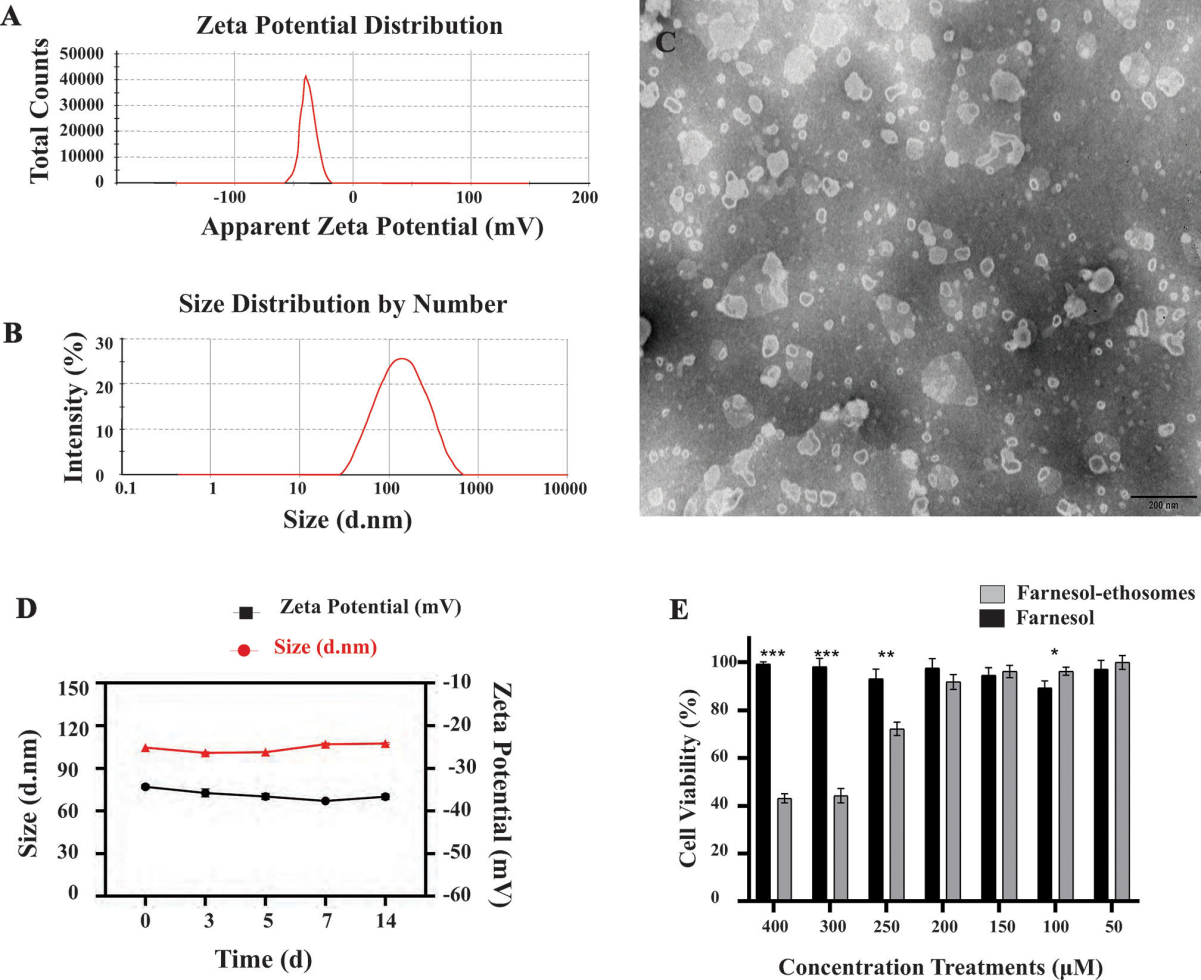
(**A**) Zeta potential of farnesol-ethosomes. (**B**) Particle-size distribution spectrum of farnesol-ethosomes. (**C**) Morphology of farnesol-ethosomes determined by TEM. Scale bar represents 200 nm. (**D**) Stability of farnesol-ethosomes dispersed at 25°C for 2 weeks. (**E**) Cell viability rates of yeast cells in response to farnesol-ethosomes and farnesol in DMSO. Yeast cells were cultured with tested components at different concentrations for 12 h. In the control group, yeast cells with ethosomes (no farnesol) or DMSO treatment were considered to reach 100% viability. Mean ± SD from three experiments at each conidiation was represented by vertical bar (***P ≤ 0.001; **P ≤ 0.01; *P ≤ 0.05).

Intensive research demonstrates that nanoparticles effectively improve drug delivery efficacy and decrease adverse drug properties through various mechanisms, such as interactions with biological membrane barriers. The size, morphology, and zeta potential of nanoparticles play a vital role in drug release and uptake in mammalian and fungal cells, making them a highly sought-after solution in medical research. In this study, farnesol-ethosomes were successfully developed by thin-film hydration method. As shown in Table 2 and Fig. 1A through B, the dynamic light scattering indicated that farnesol-ethosomes had an average particle size of 104.75 ± 2.05 nm, and the average zeta potential was –34.35 ± 0.45 mV. These results suggested that the developed nanoethosomes effectively improved skin permeability due to their negative charges and small particle size (38). The EE (%) and DL (%) of farnesol in the nanoethosomes were 82.77 ± 0.78% and 5.05 ± 0.032%, respectively, making them a highly efficient drug delivery system. Analysis of farnesol-ethosomes using TEM imaging revealed well-dispersed nanoparticles with approximately spherical shape (Fig. 1C). TEM image showed that the nanoparticles were less than 120 nm in size, consistent with scattering measurements. The stability assessment results in Fig. 1D showed no significant changes in farnesol-ethosomes particle size and zeta potential after being stored at 25°C for 2 weeks, indicating excellent storage stability.

**TABLE 2:** Therapeutic properties of farnesol-ethosomes (*n* = 3)^*a*^^,*b*^

Farnesol-ethosomes	
Zeta potential (mV)	–34.35 ± 0.45
Size (nm)	104.75 ± 2.05
EE (%)	82.77 ± 0.78
DL (%)	5.18 ± 0.032

^*a*^ All data expressed as means ± standard deviations.

^*b*^ EE: entrapment efficiency; DL: drug loading capacity.

### Antifungal activity of farnesol-ethosomes *in vitro*

An antifungal activity test was conducted in the YPD medium to determine the effect of farnesol-ethosomes and farnesol in dimethyl sulfoxide (DMSO) against yeast cells in *C. albicans in vitro*. The results are shown in Fig. 1E, indicating that the farnesol-ethosomes cell viability rate is concentration-dependent. In groups treated with 300 and 400 µM farnesol-ethosomes, the cell viability rate of yeast cells in *C. albicans* was approximately 43%. Conversely, the cell viability rate was generally 100% in groups treated with farnesol in DMSO. Farnesol-ethosomes showed increased inhibition effects on yeast cells’ growth at concentrations more than 250 µM, whereas farnesol in DMSO ineffectively inhibited cell growth in all tested concentrations. This result indicated that *C. albicans* was more tolerant to farnesol than farnesol-ethosomes.

### Biofilm growth of *C. albicans* in response to farnesol-ethosomes *in vitro*

*C. albicans* can seriously threaten human health by forming biofilms on the body surfaces during infection. These biofilms are complex communities of three-dimensional yeasts with different shapes, including a round-to-oval cell, hyphae with thin, tubal cell, and pseudohyphal with features of both yeast and hyphae (39). Hyphae formation is crucial in the infection process, and it is vital in the morphogenesis aspect of the *C. albicans* virulence since it enables the fungus to escape from immune cells and produce proteinase-disrupting tissues (40). Farnesol, a molecule that regulates the transcription factors responsible for the transition of hyphae to yeast (41), has shown promising results in controlling hyphae formation. We determine whether a change in ethosome drug delivery could decrease hyphae formation in *C. albicans* during biofilm growth *in vitro*.

**Fig 2:**
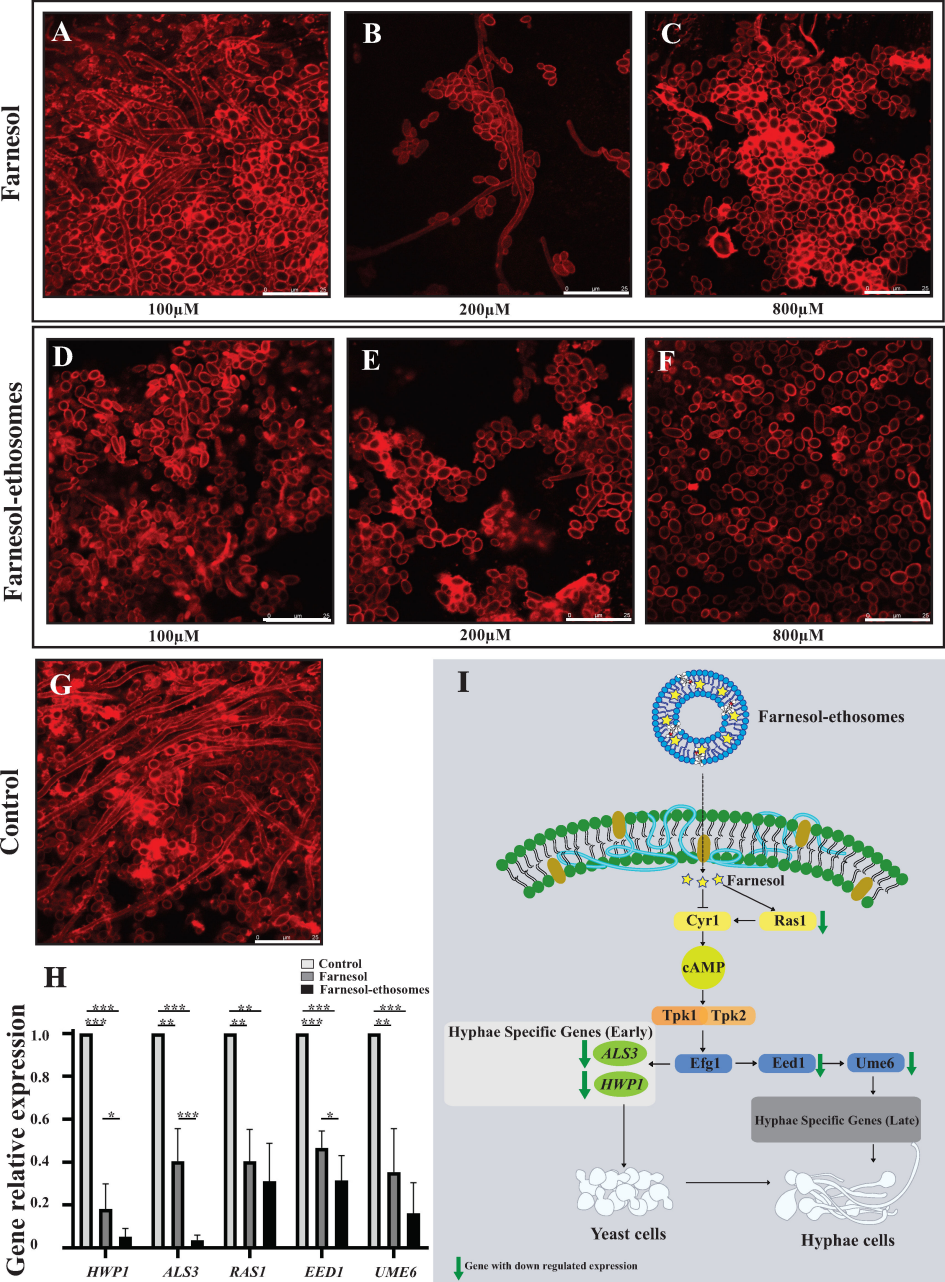
(**A–C**) The *C. albicans* cells were cultured with farnesol (from 100 to 800 µM) for 24 h in biofilm growth conditions. (**D and E**) The *C. albicans* cells strain was incubated with farnesol-ethosomes (from 100 to 800 µM) for 24 h in biofilm growth conditions. (**F**) Control group. Fluorescent images of *C. albicans* cells in biofilm growth conditions were photographed by confocal microscopy. Concanavalin A–Alexa Fluor 594 conjugate excitation generated the red fluorescent channel. All scale bars represent 25 µm. (**H**) Comparison of *HWP1*, *ALS3*, *RAS1*, *EED1*, and *UME6* gene expression pattern in response to farnesol-ethosomes and farnesol treatment (400 µM) by real-time PCR. Mean ± SD from three experiments at each conidiation was represented by vertical bar (***P ≤ 0.001; **P ≤ 0.01; *P ≤ 0.05). (**I**) Schematic of farnesol effect on signaling pathways in regulating gene expression of early and late hyphae-specific genes in *C. albicans*. Green arrows indicate genes with decreased expression in response to farnesol-ethosomes versus farnesol treatment.

However, the effectiveness of farnesol is limited due to poor drug delivery methods. Ethosomes offer a unique solution to this problem by providing a more efficient and targeted delivery of farnesol to the affected area. Ethosomal farnesol can significantly reduce the severity of *C. albicans* infections by reducing hyphae formation. This study built the matured *C. albicans* biofilm model and tested farnesol-ethosomes activity in controlling hyphae formation, depicting the results in Fig. 2. Increased drug concentration in groups treated with farnesol (Fig. 2A through C) has led to observable cell-type switching in *C. albicans*. Although confocal microscopy revealed the presence of three types of *C. albicans* cells at 100 µM farnesol treatment, alike to the control group (Fig. 2G), the proportion of hyphal cells significantly decreased. At 200 µM farnesol (Fig. 2B), the biofilm structure transitioned from a dense status into a loose one, and hyphal cells were rare but still visible. The disappearance of hyphal cells and prevalence of yeast cells was observed in groups treated with 800 µM farnesol (Fig. 2C). These results indicated that farnesol, as a quorum-sensing molecule, regulated cell-type switching from hyphal cells to yeast cells in matured biofilms. The proportion of hyphal cells is negatively correlated with the quantity of farnesol in the environment. As a result, the intensity of cell-type switching regulated by farnesol is concentration-dependent. Cell-type switching in *C. albicans* was also observed by confocal microscopy in farnesol-ethosomes treatment groups (Fig. 2D through F). Interestingly, hyphal cells were rarely observed in groups treated with 100 µM farnesol-ethosomes (Fig. 2D), similar to the observation in groups treated with 800 µM farnesol. This result was reproduced in groups treated with 200 and 800 µM farnesol-ethosomes (Fig. 2E and F). The results indicated that 200 and 800 µM farnesol-ethosomes treatments overdose in regulating cell-type switching in matured biofilms. Therefore, cell-type switching in *C. albicans* is intensively regulated in response to farnesol-ethosomes treatment versus farnesol treatment.

We investigated the critical role of gene expression in response to farnesol using real-time PCR to determine the response to tested compounds at the transcriptional level. The results shown in Fig. 2H indicate that the expression of *HWP1*, *ALS3*, *RAS1*, *EED1*, and *UME6* significantly reduced following treatment with 400 µM farnesol. Notably, Als3 and Hwp1, family members of the agglutinin-like sequence (Als), function as cell wall adhesins in *C. albicans*. Hwp1 is also expressed on the *C. albicans* hyphal cell surface. *HWP1* and *ALS3* belonging to early hyphae-specific genes are essential for *C. albicans* cell-cell interactions during the initiation step of hyphae formation (42, 43). Hyphae initiation and long-term maintenance are equally required for hyphal growth. The activation of *UME6* expression through Eed1 is required for long-term maintenance of hyphae-specific gene expression (44, 45).

Farnesol binds to the cyclase domain of the adenylyl cyclase Cyr1, directly affecting intracellular cAMP levels and promoting the cleavage of Ras1 to deactivate Cyr1 indirectly. Moreover, farnesol’s inhibitory function on the Ras1-cAMP-PKA cascade helps maintain yeast cell formation. The transcriptional activators such as Efg1, Eed1, and Ume6 are involved in the farnesol response (18, 46, 47), but their specific contributions remain largely unknown. A detailed representation of the transduction of farnesol leading to the inhibition of hyphae formation can be seen in Fig. 2I. These results suggest that farnesol greatly inhibits the expression of genes involving hyphae formation and maintenance in *C. albicans*.

### Antifungal activity of farnesol-ethosomes in the treatment of cutaneous candidiasis

**Fig 3:**
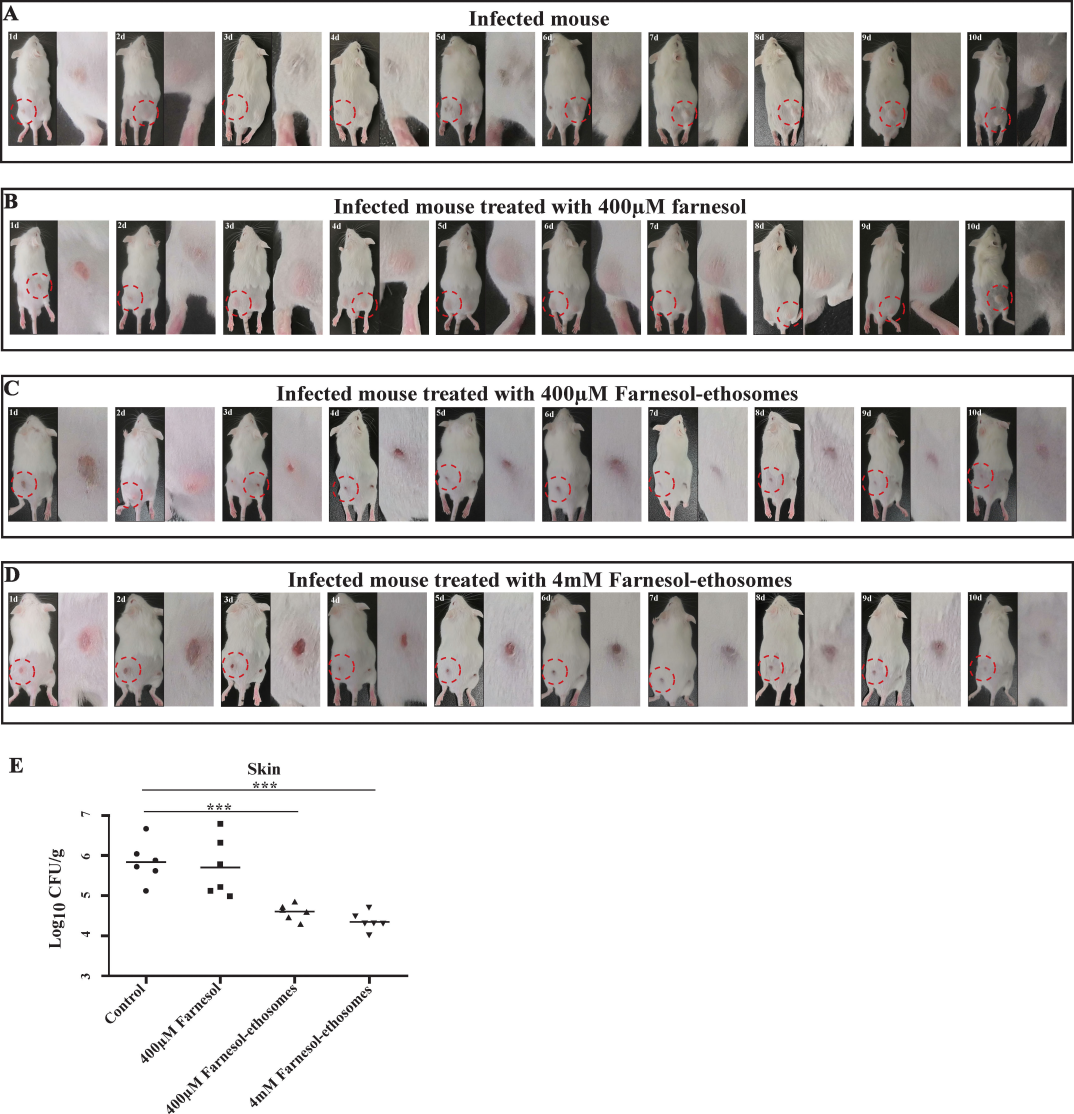
(**A–D**)Twenty-four Kunming mice were randomly divided into four groups and injected subcutaneously with yeast cells in *C. albicans*. Transdermal treatment of 400 µM farnesol, 400 µM farnesol-ethosomes, 4 mM farnesol-ethosomes, and PBS was conducted in each group from day 1 to day 10. The lesion sites in each mouse were photographed daily until day 10. (**E**) A fungal burden test was performed on lesion sites of mouse skin. Transdermal treatment of both farnesol-ethosomes dramatically inhibited *C. albicans* proliferation in mouse skin versus farnesol (***P ≤ 0.001).

A mouse model of cutaneous candidiasis induced by *C. albicans* was conducted to evaluate farnesol-ethosomes’ efficacy *in vivo*. The study involved the male Kunming mice subcutaneously infected with *C. albicans* for 3 days *in situ* to establish an infected model. After this, 24 mice with *C. albicans* cells’ infection were randomly divided into four groups and treated respectively with PBS, 400 µM farnesol, 400 µM farnesol-ethosomes, and 4 mM farnesol-ethosomes via transdermal drug delivery. The treatment was initiated on Day 1, and lesion sites were photographed daily using a camera. On day 10 post-treatment, the mice were sacrificed, and the skin tissues at the lesion site were dissected and homogenized for fungal burden tests. Fig. 3 presents the results. In the control group (Fig. 3A), erythematous papules appeared at the lesion site on day 1 and visible nodules on day 6. The nodules grew in size and were apparently on day 10, indicating the formation of lymphocutaneous at primary lesion sites by subcutaneous infection. The results obtained in the 400 µM farnesol treatment group were similar (Fig. 3B). However, 400 µM farnesol treatment caused no apparent changes in lymphocutaneous. In two farnesol-ethosomes treatment groups (Fig. 3C and D), erythematous papules were also observed at the lesion site on day 1. Interestingly, ulcers appeared on day 3, and the open wounds continued to heal until closure. Only a slight mark was observed at the lesion site on day 10. The disappearance of lymphocutaneous at the lesion site and intact skin structure show that farnesol-ethosomes treatment almost healed the infected skin. There was no significant difference between the two farnesol-ethosomes treatment groups. It seems that the escalation in dosage dose not yield a substantial enhancement in the therapeutic efficacy. We also observed a noticeable reduction in fungal burdens between farnesol-ethosomes treatment groups and the control group (Fig. 3E). We observed a striking significant reduction in lower numbers of CFU in the skins of both farnesol-ethosomes treatment groups compared to the farnesol treatment group, indicating a 12.4-fold decrease in 400 µM farnesol-ethosomes treatment group and 31-fold in 4 mM farnesol-ethosomes treatment group. However, the numbers of CFU in the 4 mM farnesol-ethosomes treatment group were only 2.5-fold higher than that in the 400 µM farnesol-ethosomes treatment group. These results suggest that farnesol-ethosomes’ antifungal activity significantly improves in treating cutaneous candidiasis using transdermal administration in mice versus farnesol in ordinary drugs. The farnesol-ethosomes fungistatic effect is highly correlated with encapsulation by nanoparticles.

### C6-ethosomes uptake *in vitro* and *in vivo*

**Fig 4:**
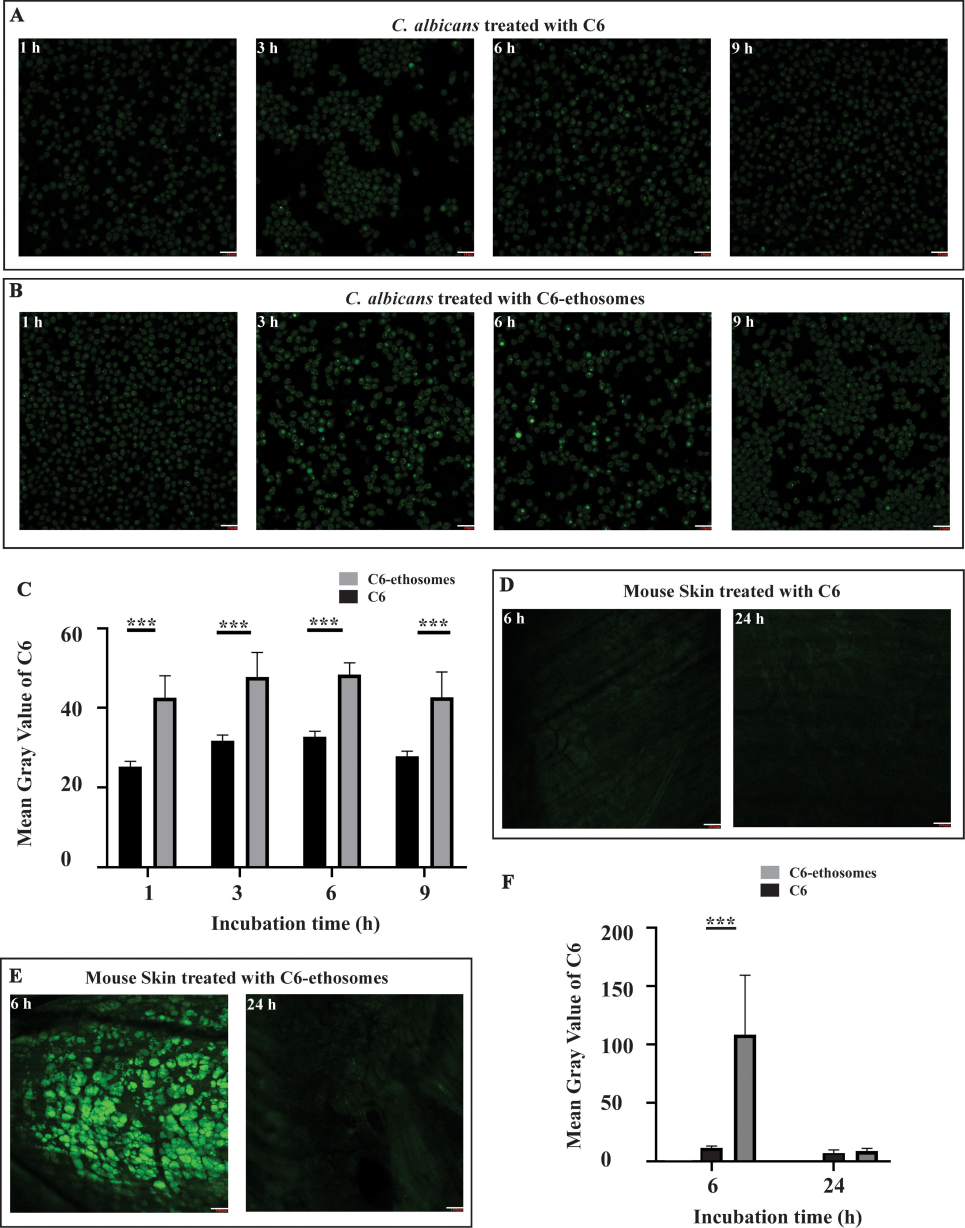
(**A and B**) Yeast cells in *C. albicans* were treated with C6-ethosomes or C6 for 1, 3, 6, and 9 h. Fluorescent images of yeast cells in *C. albicans* were photographed by Leica TCS SPE. Excitation of florescence dye C6 generated green fluorescent channel. All scale bars are 10 µm. (**C**) Mean gray values of C6-ethosomes or C6 in *C. albicans* yeast cells were measured quantitatively by ImageJ at time points (1, 3, 6 and 9 h). (**D and E**) Transdermal treatment of C6-ethosomes or C6 for 6 and 24 h was performed in a mouse model of cutaneous candidiasis induced by *C. albicans*. The specific mouse skin was dissected off muscle and photographed by Leica TCS SPE. Excitation of florescence dye C6 generated green fluorescent channel. All scale bars are 100 µm. (**F**) Mean gray values of C6-ethosomes or C6 in mouse skins were measured quantitatively by ImageJ at time points (6 and 24 h). Mean ± SD from replicated experiments at each conidiation was represented by vertical bar (***P ≤ 0.001).

Drug molecules’ cellular uptake efficacy was commonly evaluated using confocal microscopy via fluorescent probes such as C6 (48, 49). We quantitatively examined *C. albicans* cells uptake C6-ethosomes by confocal microscopy to determine the effect of ethosomes on drug delivery efficacy *in vitro* Fig. 4A shows C6 uptake into yeast cells at different time courses in *C. albicans*. The result indicated that the fluorescence intensity of C6 reached the maximum in yeast cells at 3 h. The maximum level of the fluorescence intensity lasted until 6 h and decreased at 9 h. These results suggest that the fluorescence intensity of C6 varies with time courses. In the C6-ethosomes treatment group (Fig. 4B), changes in fluorescence intensity of C6 similarly depended on time courses. However, the maximum fluorescence intensity appearing in the C6-ethosomes treatment group was more than that in the C6 treatment group, precisely at 3 and 6 h. These results suggest that the rate of fluorescent probe C6 uptake in yeast cells varies between C6-ethosomes and C6 treatment groups. Furthermore, the fluorescence signals in each group were quantified as the mean gray value as determined by ImageJ (Fig. 4C). The mean gray value in the C6-ethosomes treatment group reached its peak at 6 h, which is 47.7% higher than the highest value of C6 at 3 h. The improved mean gray value in the C6-ethosomes treatment group could be attributed to the increased accumulation of the fluorescent probes in cytoplasm. These results indicated that the drug delivery efficacy of C6 was improved in yeast cells by ethosomes *in vitro*.

We randomly divided 30 mice into three groups and treated them with C6-ethosomes, C6, and PBS buffer on the skin through transdermal administration. Our goal is to determine the drug delivery efficacy of ethosomes *in vivo*, At 6 and 24 h post-treatment, five mice in each group were sacrificed, and the skin tissues with test compound treatment were dissected and photographed using confocal microscopy. Results in the control group were not shown. In the C6 treatment group (Fig. 4D), the fluorescence intensity of C6 was not obviously detected from mouse skin after 6 and 24 h treatment. However, in the C6-ethosomes treatment group (Fig. 4E), the fluorescence intensity of C6 was visible at 6 h and significantly declined at 24 h. Additionally, the fluorescence signals were quantified in each group using the mean gray value by ImageJ (Fig. 4F). The mean gray value in the C6-ethosomes treatment group at 6 h was almost 10-fold greater than that in C6. Our findings indicate no significant difference between the two groups at 24 h. However, our study showed that C6-ethosomes had a remarkable drug delivery effectiveness through transdermal administration in mice compared to ordinary drug C6.

### Cytotoxicity of farnesol-ethosomes *in vivo* and *in vitro*

**Fig 5:**
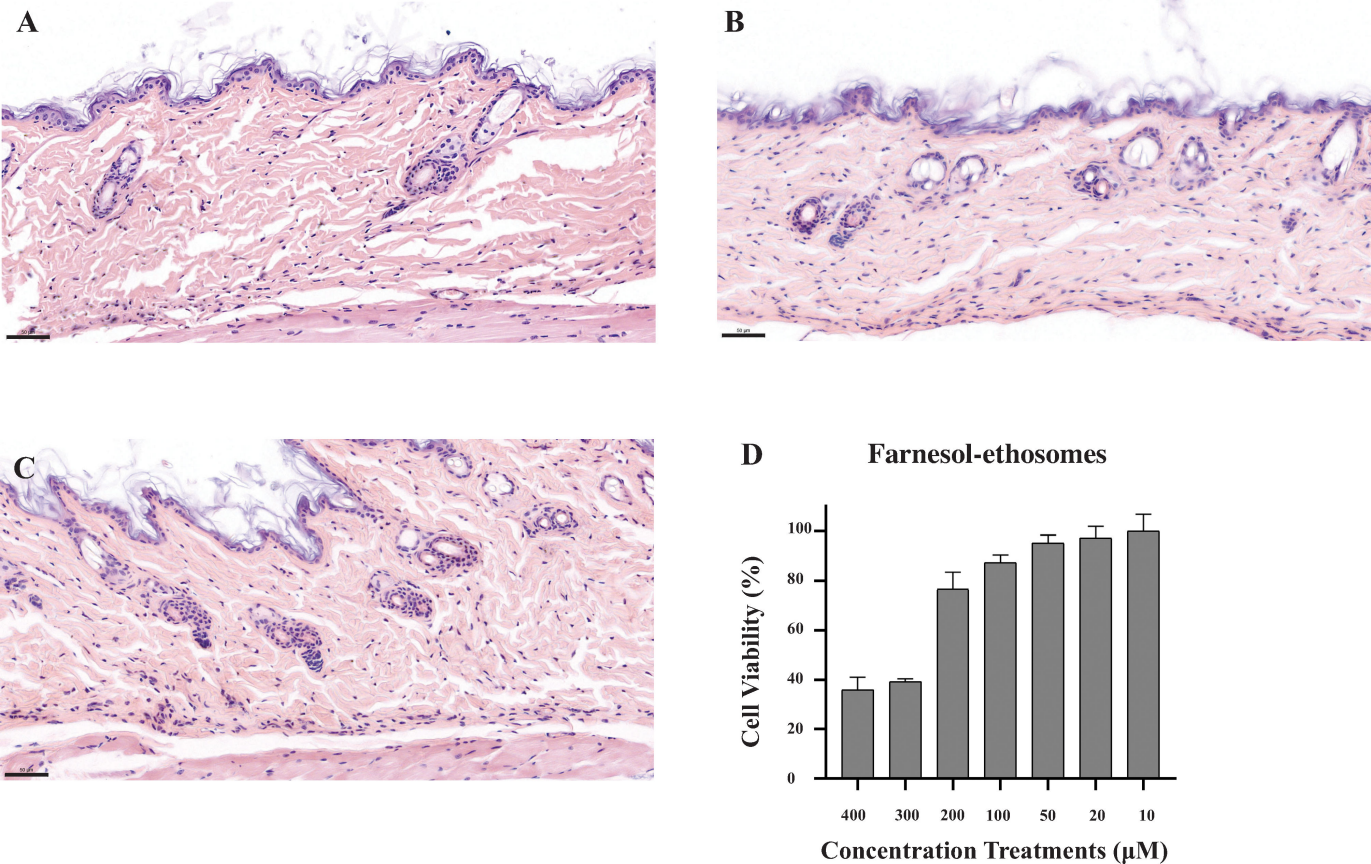
The effects of farnesol-ethosomes on skin tissue were evaluated in a mouse model. The mice skins treated with PBS (**A**), 400 µM farnesol (**B**), and 400 µM farnesol-ethosomes (**C**) with H&E staining were examined by pathological sections. All scale bars are 50 µm. (**D**) Viability rates of the 293T cells in response to farnesol-ethosomes treatment *in vitro*. Mean ± SD from three experiments was represented by vertical bar.

**Fig 6:**
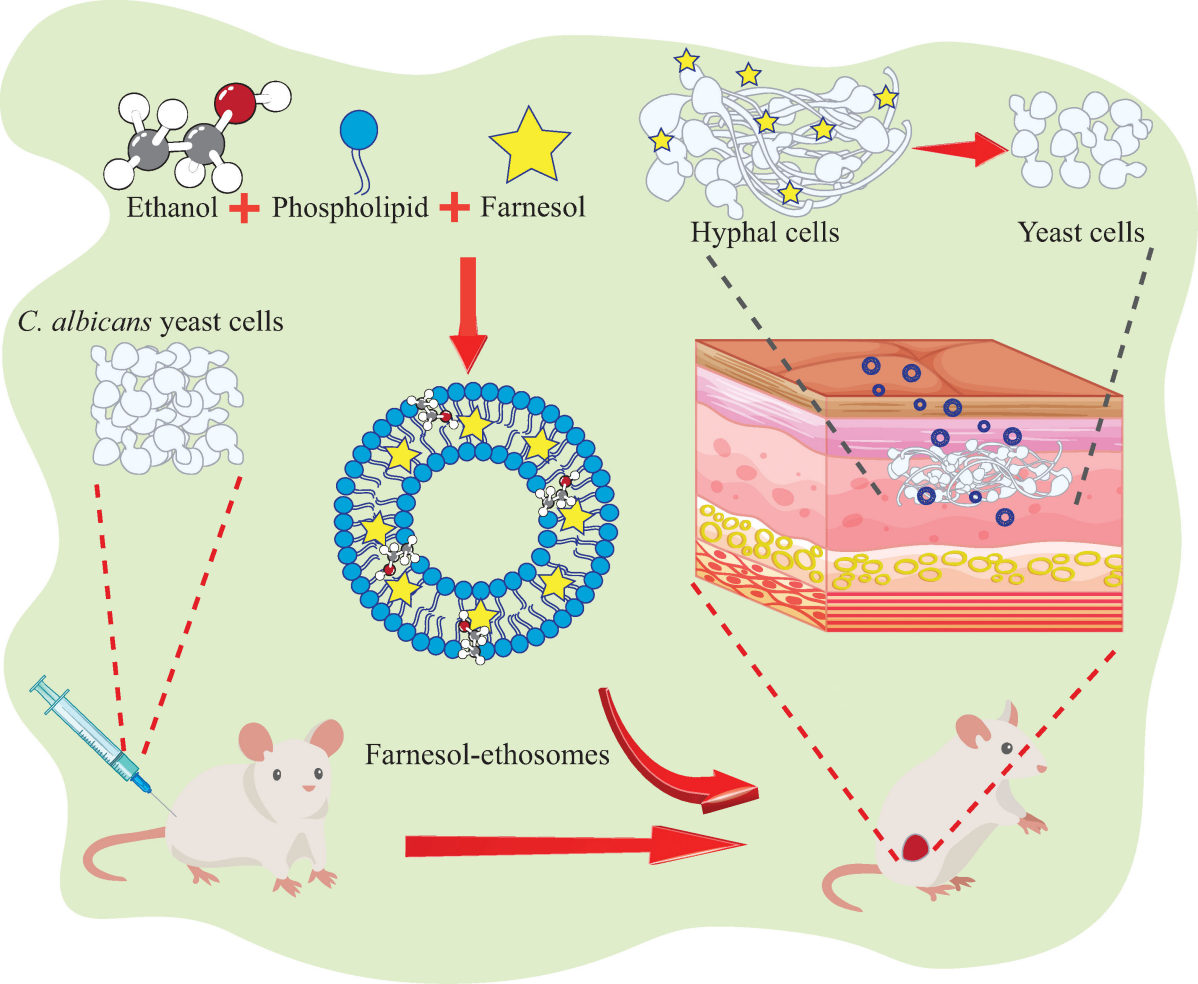
Schematic illustration of farnesol-ethosomes in the transdermal treatment of cutaneous candidiasis induced by *C. albicans* in mice. Farnesol-ethosomes are prepared by thin-film hydration method. Treatment of farnesol-ethosomes greatly promotes the switching from hyphae cells to yeast cells and significantly reduces *C. albicans* proliferation in mouse skin.

The therapeutic effect of farnesol-ethosomes will be exaggerated if the toxicity is ignored partly, even if transdermal delivery of farnesol-ethosomes against cutaneous candidiasis is limited in specific areas of the skin. A mouse model was used to study the effects of farnesol-ethosomes on skin tissue and evaluate the cytotoxicity of farnesol-ethosomes *in vivo*. This study randomly divided 15 male mice into three groups and served them respectively with PBS, 400 µM farnesol, and 400 µM farnesol-ethosomes through transdermal administration for 10 days. Mice were sacrificed, and skin tissues were dissected for pathological sections. H&E staining of skin (Fig. 5A through C) shows no noticeable pathological changes among the control group (Fig. 5A), farnesol treatment group (Fig. 5B), and farnesol-ethosomes treatment group (Fig. 5C). Three layers in skin structure, including epidermis, dermis, and subcutaneous tissue, were intact, and noticeable lesions were not observed. Their results indicated farnesol-ethosomes treatment by transdermal administration in mice would be safe. A cytotoxicity assay was performed in mammalian cells to test the safety of farnesol-ethosomes *in vitro*. Then, 293T cells received farnesol-ethosomes treatment, and the cell viability rate was calculated, as shown in Fig. 5D. The cell viability rate of 293T cells in response to farnesol-ethosomes was concentration-dependent. Treatment with farnesol-ethosomes less than 100 µM resulted in a cell viability rate of approximately 95% in 293T cells, while it decreased to approximately 36% at 400 µM farnesol-ethosomes treatment. These findings suggest that farnesol-ethosomes treatment would be safe at concentrations less than 100 µM *in vitro*. However, the farnesol-ethosomes dosage for treating cutaneous candidiasis should be carefully determined. Considering the antifungal activity of farnesol-ethosomes *in vivo*, 400 µM farnesol-ethosomes are recommended in treating mouse models of cutaneous candidiasis induced by *C. albicans*. The comprehensive findings are succinctly presented in Fig. 6.

## DISCUSSION

This study successfully developed farnesol-ethosomes as a novel treatment for cutaneous candidiasis induced by *C. albicans* in mice. The farnesol-ethosomes were found to be spherical with proper particle size, exhibited great EE and DL, and showed good storage stability at 25°C for 2 weeks. Farnesol-ethosomes showed an increased inhibition effect against yeast cells at concentrations more than 300 µM in *C. albicans*. Although the most remarkable ability of farnesol is to affect *C. albicans* morphology with no effects on yeast cells’ growth in *C. albicans*, increased ROS production and disruption of mitochondrial function have been proposed to cause mitochondrial dysfunction and cell death induced by farnesol in *C. albicans* (50, 51). Farnesol-ethosomes increase antifungal activity compared to farnesol’s conventional form, most likely due to the increase in solubility and stability by nanoencapsulation.

During biofilm growth, farnesol-ethosomes promoted the switching from hyphae cells to yeast cells by decreasing the expression of hyphae-related genes in *C. albicans*. Expressions of certain genes, such as *HWP1* and *ALS3*, responsible for hyphae formation and maintenance, were more inhibited in response to a 400 µM farnesol-ethosomes treatment. Farnesol-ethosomes seem to have a more significant inhibitory effect on genes participating in hyphae formation and maintenance at the transcriptional level. Recent studies have suggested that the sub-apical collar region in filamentous fungi exhibits a higher concentration of endocytosis-related activities and is essential for hyphal growth. It is possible that the incorporation of ethosomes could potentially enhance the uptake process during biofilm formation in *C. albicans* (52, 53). Therefore, encapsulation of farnesol in ethosomes improves the effect of farnesol in regulating morphogenesis and controlling cell growth in *C. albicans*.

Surprisingly, farnesol-ethosomes exhibited enhanced antifungal activity *in vivo*. Transdermal administration of farnesol-ethosomes in mice significantly reduced symptoms of cutaneous candidiasis induced by *C. albicans*. Fungal burden analysis demonstrated a dramatic decline in the quantity of *C. albicans* cells in farnesol-ethosomes treatment groups compared to farnesol alone, strongly suggesting improved therapeutic efficacy. The development of ethosomes on drug delivery efficacy was evaluated using fluorescent probe C6 in *C. albicans* cells and mice model. Encapsulation by ethosomes proved higher efficacy *in vitro* and *in vivo* drug delivery than traditional dosages. The limited drug delivery efficacy of C6 in ordinary drugs, due to the barrier in stratum corneum in the epidermis, can be associated with limitation in small, lipophilic molecules by passive diffusion (54). However, the vesicle structure formed by ethosomes directly penetrates the stratum corneum. Due to the higher concentration of alcohol in phospholipid layer, ethosomes have better flexibility and membrane fluidity than the other nanoparticles such as liposomes. Alcohol is also able to reduce the critical temperature of lipid in stratum corneum and increase its fluidity. Therefore, the penetration depth by ethosomes is much deeper, and the drug transdermal efficiency is significantly improved (55). Meanwhile, the dense and structure of the stratum corneum in skin is disrupted by phospholipids in ethosomes fusing with the stratum corneum lipids. The drug is released from the vesicles and penetrates into the skin (56). Therefore, ethosomes can be a promising nanocarrier for farnesol in transdermal delivery. More importantly, farnesol-ethosomes’ safety was verified in mice and 293T cells. Our study demonstrated the superior features of farnesol-ethosomes in treating cutaneous candidiasis induced by *C. albicans*. It also provides clinical treatment of dermatomycoses with a novel insight.
